# Supplementary material for: Benzoic Acid Metabolism and Lipopolysaccharide Synthesis of Intestinal Microbiome Affects the Health of Ruminants under Free-Range and Captive Mode
Source: Life (Basel). 2022 Jul 18;12(7):1071. doi: 10.3390/life12071071 (PMC9317595; doi:10.3390/life12071071)
Supplement: Supplementary file 1 [file life-12-01071-s001.zip › life-1781662-supplementary.pptx]

## Slide 1
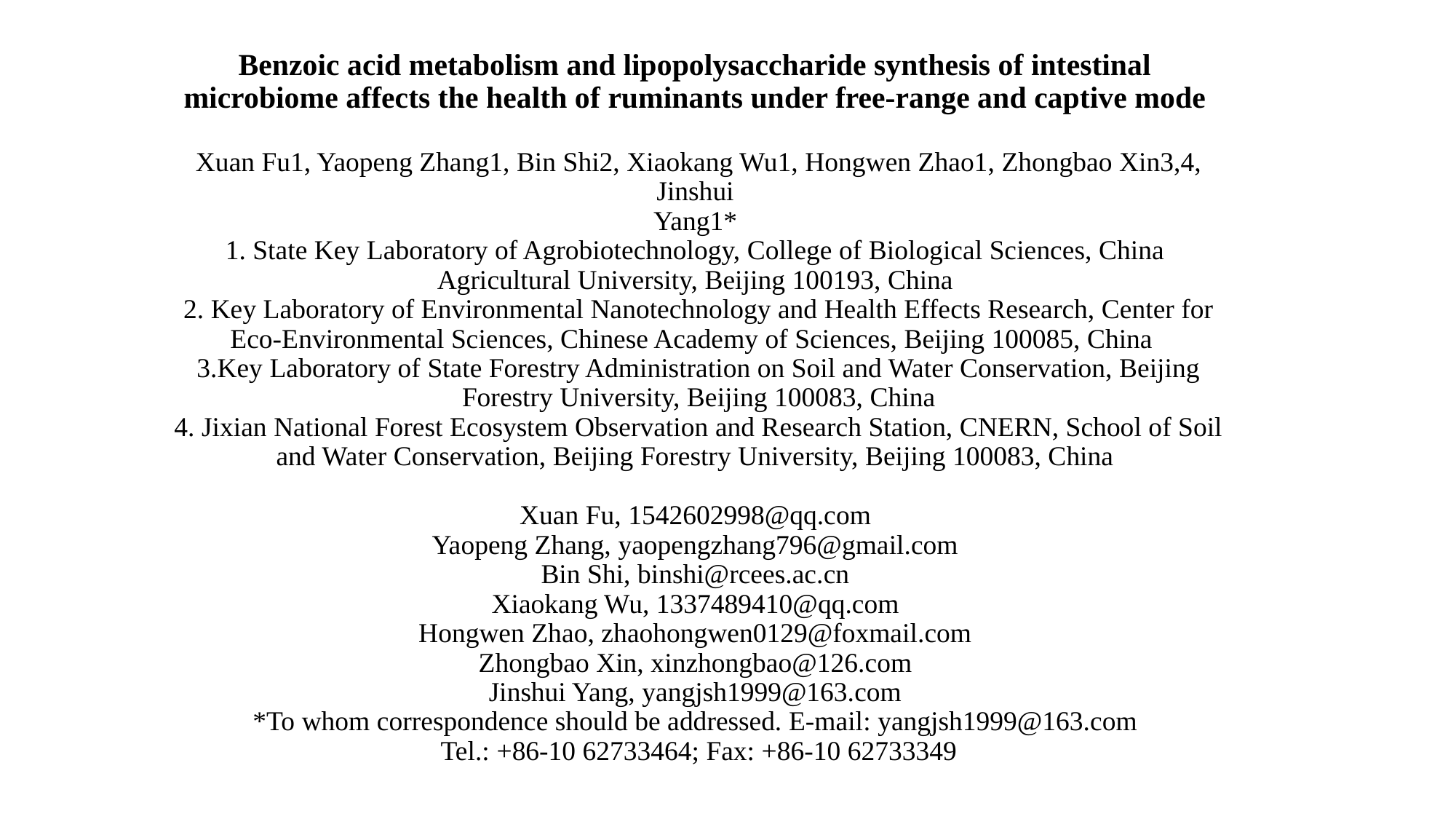

# Benzoic acid metabolism and lipopolysaccharide synthesis of intestinal microbiome affects the health of ruminants under free-range and captive mode Xuan Fu1, Yaopeng Zhang1, Bin Shi2, Xiaokang Wu1, Hongwen Zhao1, Zhongbao Xin3,4, Jinshui Yang1* 1. State Key Laboratory of Agrobiotechnology, College of Biological Sciences, China Agricultural University, Beijing 100193, China 2. Key Laboratory of Environmental Nanotechnology and Health Effects Research, Center for Eco-Environmental Sciences, Chinese Academy of Sciences, Beijing 100085, China 3.Key Laboratory of State Forestry Administration on Soil and Water Conservation, Beijing Forestry University, Beijing 100083, China 4. Jixian National Forest Ecosystem Observation and Research Station, CNERN, School of Soil and Water Conservation, Beijing Forestry University, Beijing 100083, China Xuan Fu, 1542602998@qq.com Yaopeng Zhang, yaopengzhang796@gmail.com Bin Shi, binshi@rcees.ac.cn Xiaokang Wu, 1337489410@qq.com Hongwen Zhao, zhaohongwen0129@foxmail.com Zhongbao Xin, xinzhongbao@126.com Jinshui Yang, yangjsh1999@163.com *To whom correspondence should be addressed. E-mail: yangjsh1999@163.com Tel.: +86-10 62733464; Fax: +86-10 62733349

## Slide 2
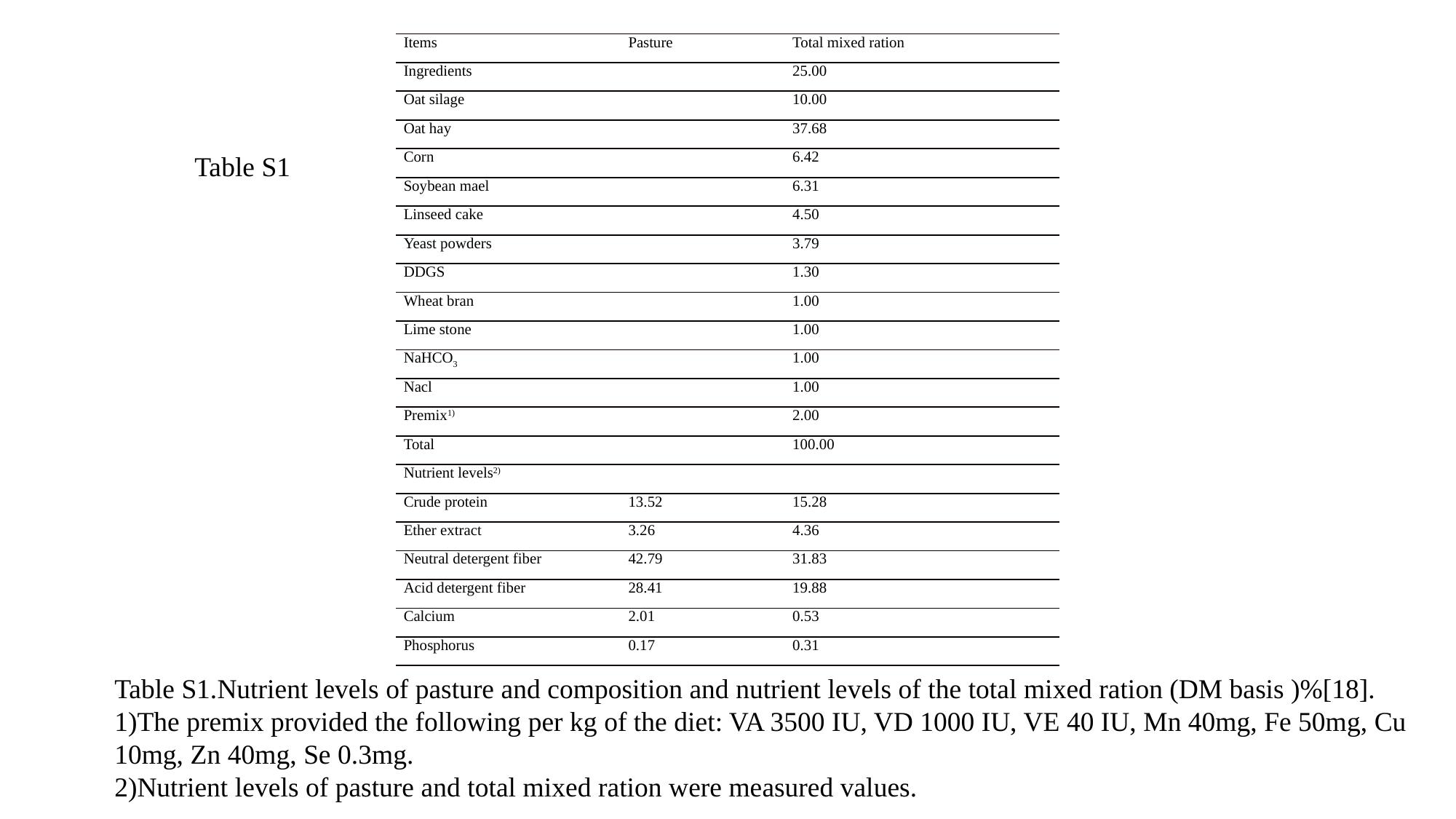

| Items | Pasture | Total mixed ration |
| --- | --- | --- |
| Ingredients | | 25.00 |
| Oat silage | | 10.00 |
| Oat hay | | 37.68 |
| Corn | | 6.42 |
| Soybean mael | | 6.31 |
| Linseed cake | | 4.50 |
| Yeast powders | | 3.79 |
| DDGS | | 1.30 |
| Wheat bran | | 1.00 |
| Lime stone | | 1.00 |
| NaHCO3 | | 1.00 |
| Nacl | | 1.00 |
| Premix1) | | 2.00 |
| Total | | 100.00 |
| Nutrient levels2) | | |
| Crude protein | 13.52 | 15.28 |
| Ether extract | 3.26 | 4.36 |
| Neutral detergent fiber | 42.79 | 31.83 |
| Acid detergent fiber | 28.41 | 19.88 |
| Calcium | 2.01 | 0.53 |
| Phosphorus | 0.17 | 0.31 |
Table S1
Table S1.Nutrient levels of pasture and composition and nutrient levels of the total mixed ration (DM basis )%[18].
1)The premix provided the following per kg of the diet: VA 3500 IU, VD 1000 IU, VE 40 IU, Mn 40mg, Fe 50mg, Cu 10mg, Zn 40mg, Se 0.3mg.
2)Nutrient levels of pasture and total mixed ration were measured values.

## Slide 3
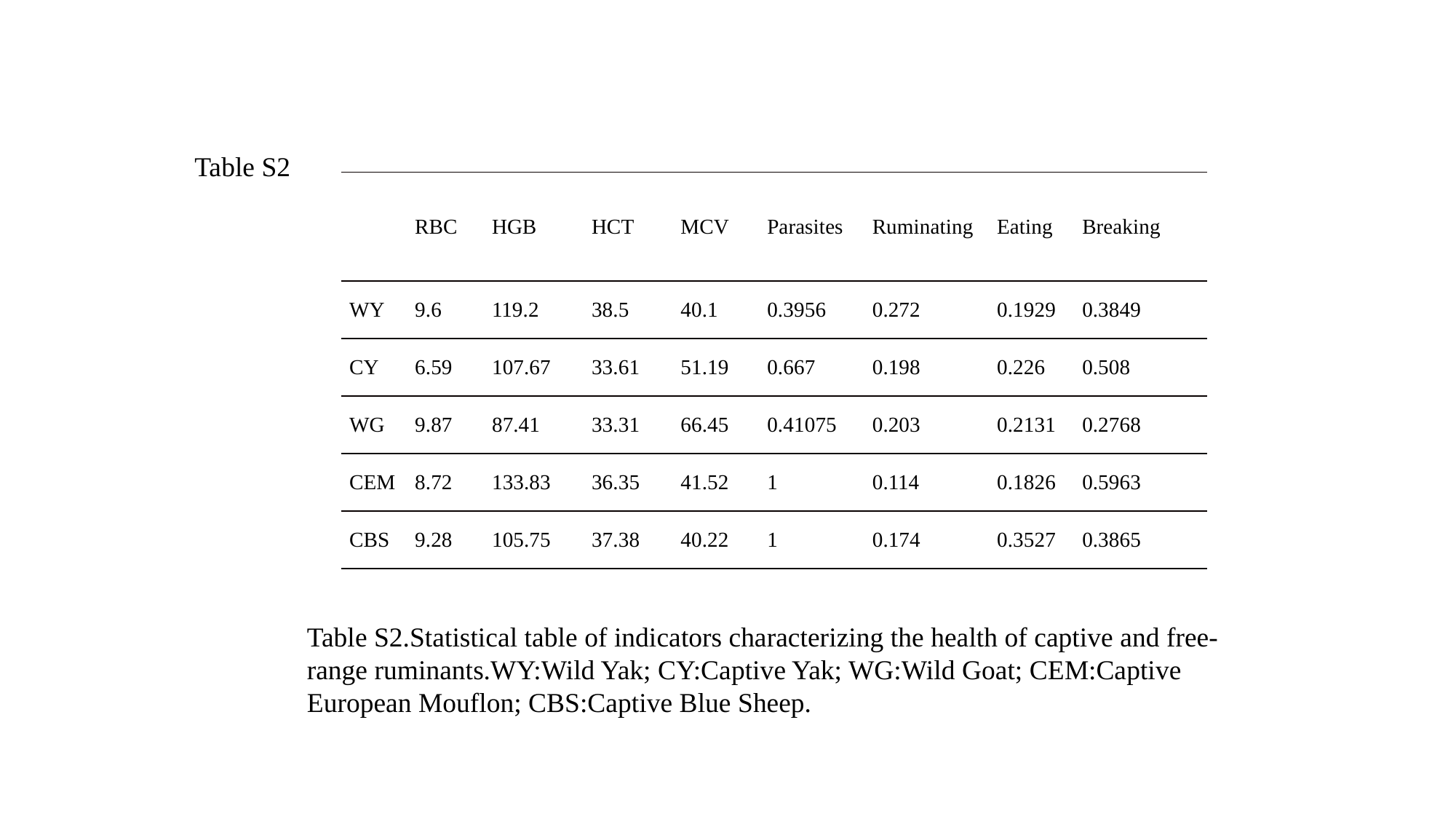

Table S2
| | RBC | HGB | HCT | MCV | Parasites | Ruminating | Eating | Breaking |
| --- | --- | --- | --- | --- | --- | --- | --- | --- |
| WY | 9.6 | 119.2 | 38.5 | 40.1 | 0.3956 | 0.272 | 0.1929 | 0.3849 |
| CY | 6.59 | 107.67 | 33.61 | 51.19 | 0.667 | 0.198 | 0.226 | 0.508 |
| WG | 9.87 | 87.41 | 33.31 | 66.45 | 0.41075 | 0.203 | 0.2131 | 0.2768 |
| CEM | 8.72 | 133.83 | 36.35 | 41.52 | 1 | 0.114 | 0.1826 | 0.5963 |
| CBS | 9.28 | 105.75 | 37.38 | 40.22 | 1 | 0.174 | 0.3527 | 0.3865 |
Table S2.Statistical table of indicators characterizing the health of captive and free-range ruminants.WY:Wild Yak; CY:Captive Yak; WG:Wild Goat; CEM:Captive European Mouflon; CBS:Captive Blue Sheep.

## Slide 4
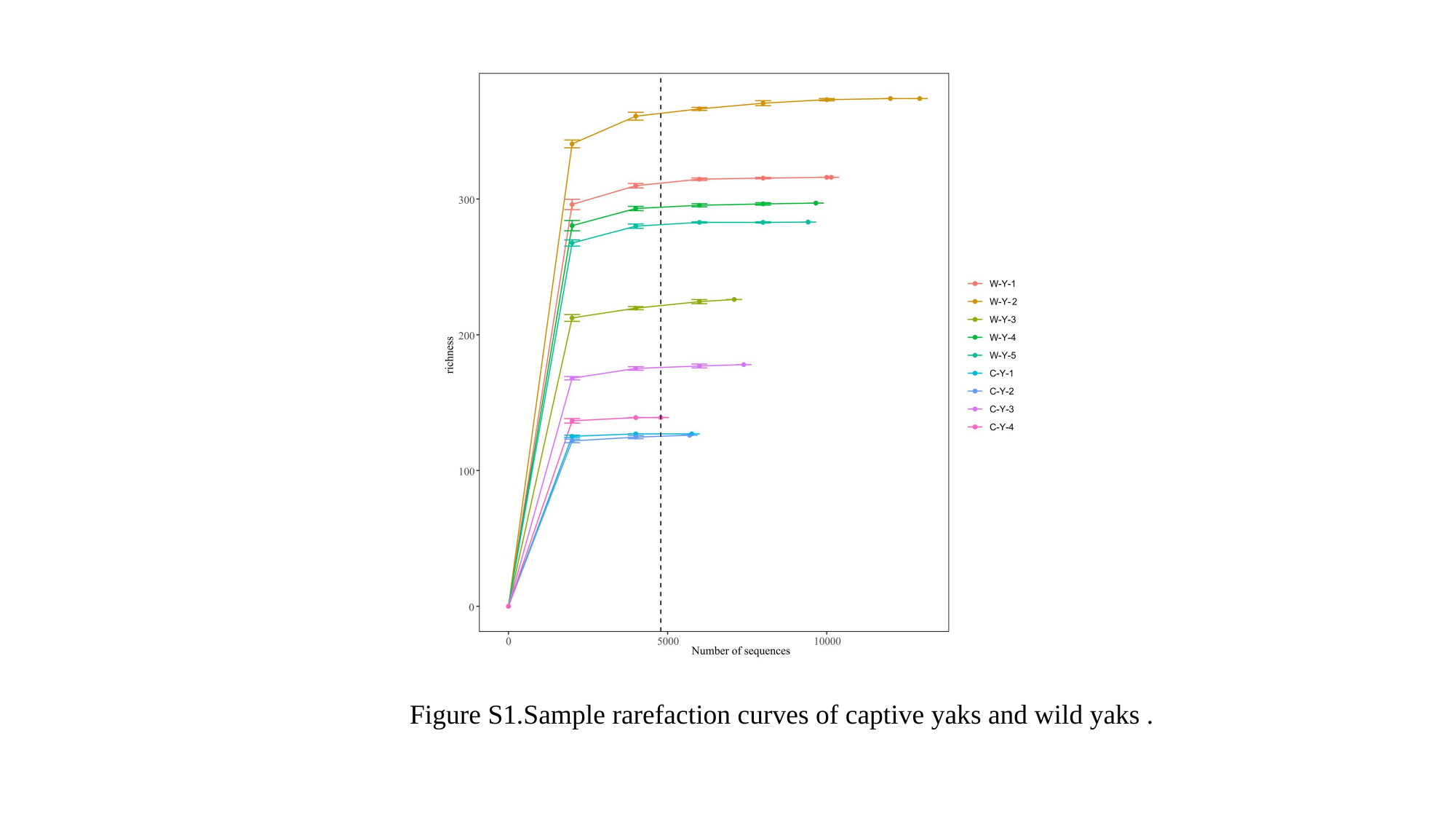

Figure S1.Sample rarefaction curves of captive yaks and wild yaks .

## Slide 5
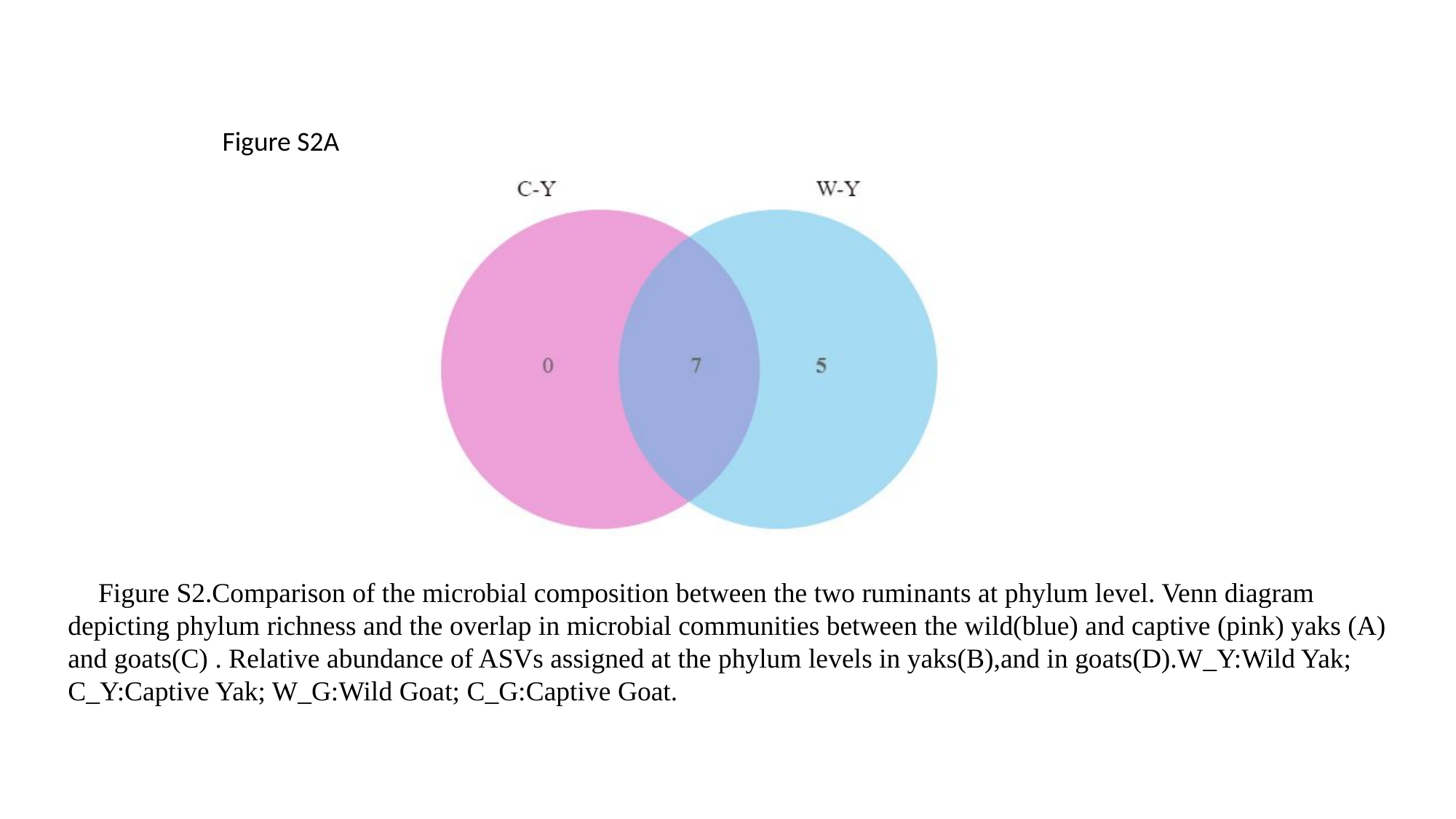

Figure S2A
Figure S2.Comparison of the microbial composition between the two ruminants at phylum level. Venn diagram depicting phylum richness and the overlap in microbial communities between the wild(blue) and captive (pink) yaks (A) and goats(C) . Relative abundance of ASVs assigned at the phylum levels in yaks(B),and in goats(D).W_Y:Wild Yak; C_Y:Captive Yak; W_G:Wild Goat; C_G:Captive Goat.

## Slide 6
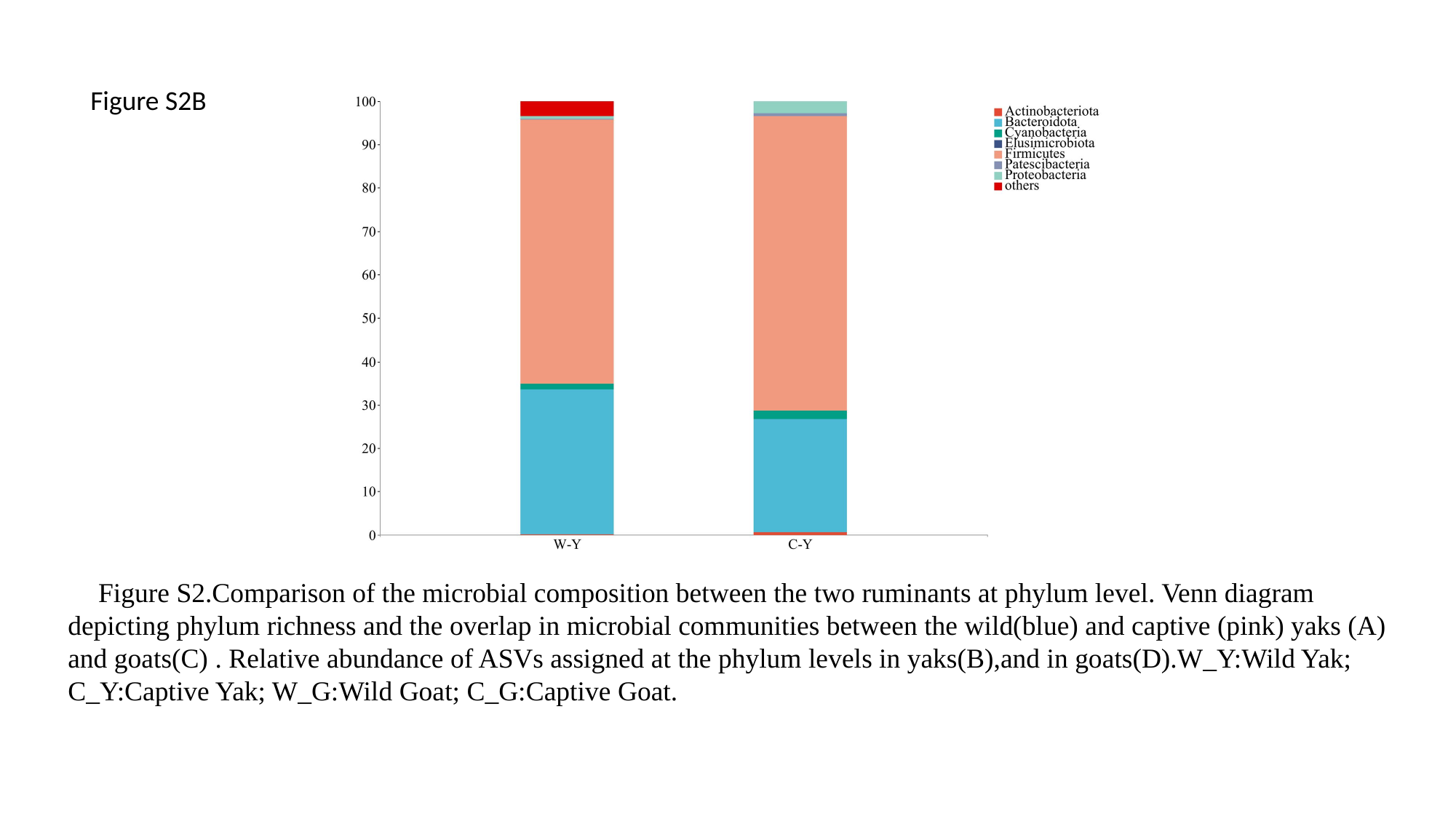

Figure S2B
Figure S2.Comparison of the microbial composition between the two ruminants at phylum level. Venn diagram depicting phylum richness and the overlap in microbial communities between the wild(blue) and captive (pink) yaks (A) and goats(C) . Relative abundance of ASVs assigned at the phylum levels in yaks(B),and in goats(D).W_Y:Wild Yak; C_Y:Captive Yak; W_G:Wild Goat; C_G:Captive Goat.

## Slide 7
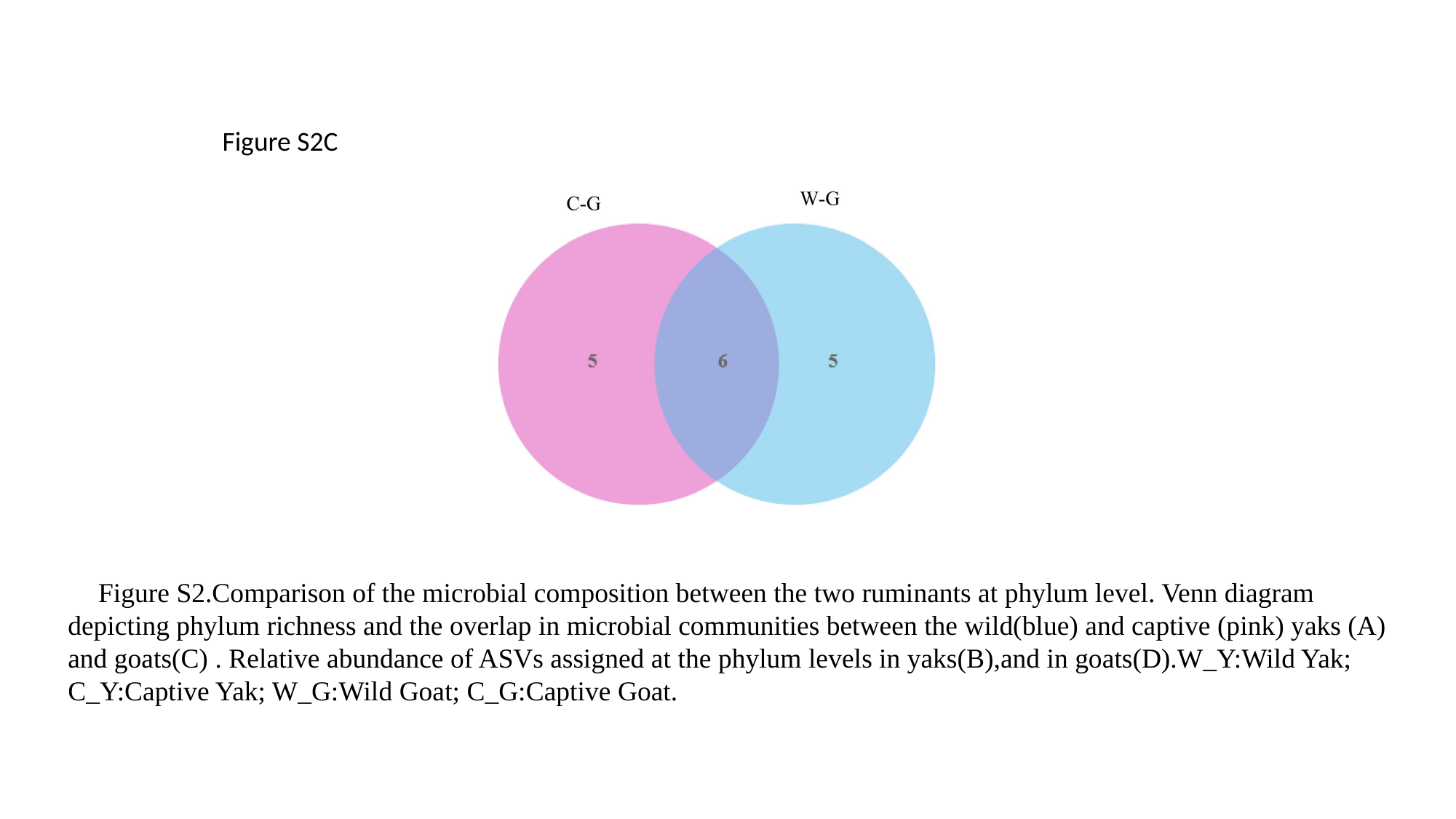

Figure S2C
Figure S2.Comparison of the microbial composition between the two ruminants at phylum level. Venn diagram depicting phylum richness and the overlap in microbial communities between the wild(blue) and captive (pink) yaks (A) and goats(C) . Relative abundance of ASVs assigned at the phylum levels in yaks(B),and in goats(D).W_Y:Wild Yak; C_Y:Captive Yak; W_G:Wild Goat; C_G:Captive Goat.

## Slide 8
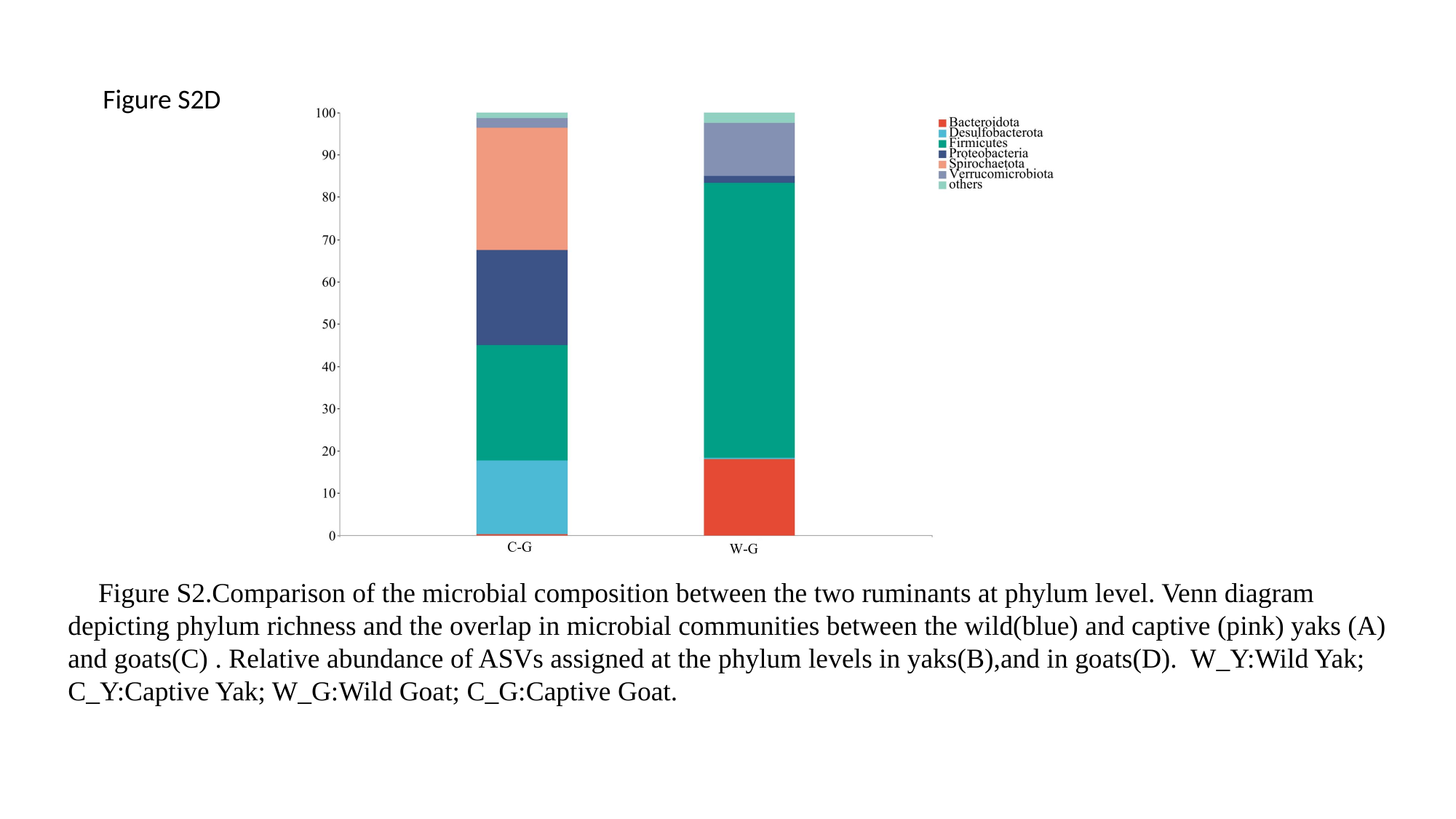

Figure S2D
Figure S2.Comparison of the microbial composition between the two ruminants at phylum level. Venn diagram depicting phylum richness and the overlap in microbial communities between the wild(blue) and captive (pink) yaks (A) and goats(C) . Relative abundance of ASVs assigned at the phylum levels in yaks(B),and in goats(D). W_Y:Wild Yak; C_Y:Captive Yak; W_G:Wild Goat; C_G:Captive Goat.

## Slide 9
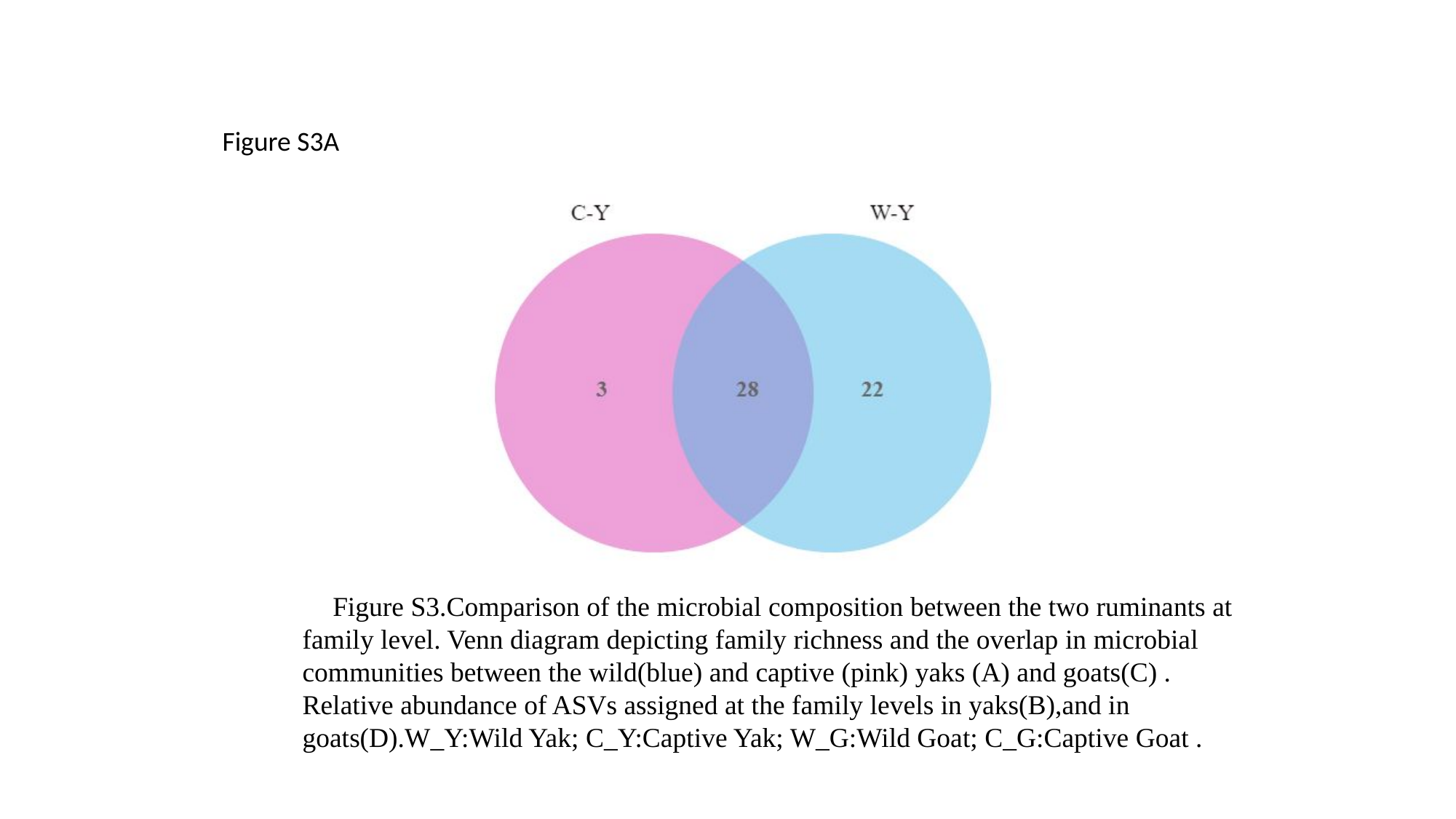

Figure S3A
Figure S3.Comparison of the microbial composition between the two ruminants at family level. Venn diagram depicting family richness and the overlap in microbial communities between the wild(blue) and captive (pink) yaks (A) and goats(C) . Relative abundance of ASVs assigned at the family levels in yaks(B),and in goats(D).W_Y:Wild Yak; C_Y:Captive Yak; W_G:Wild Goat; C_G:Captive Goat .

## Slide 10
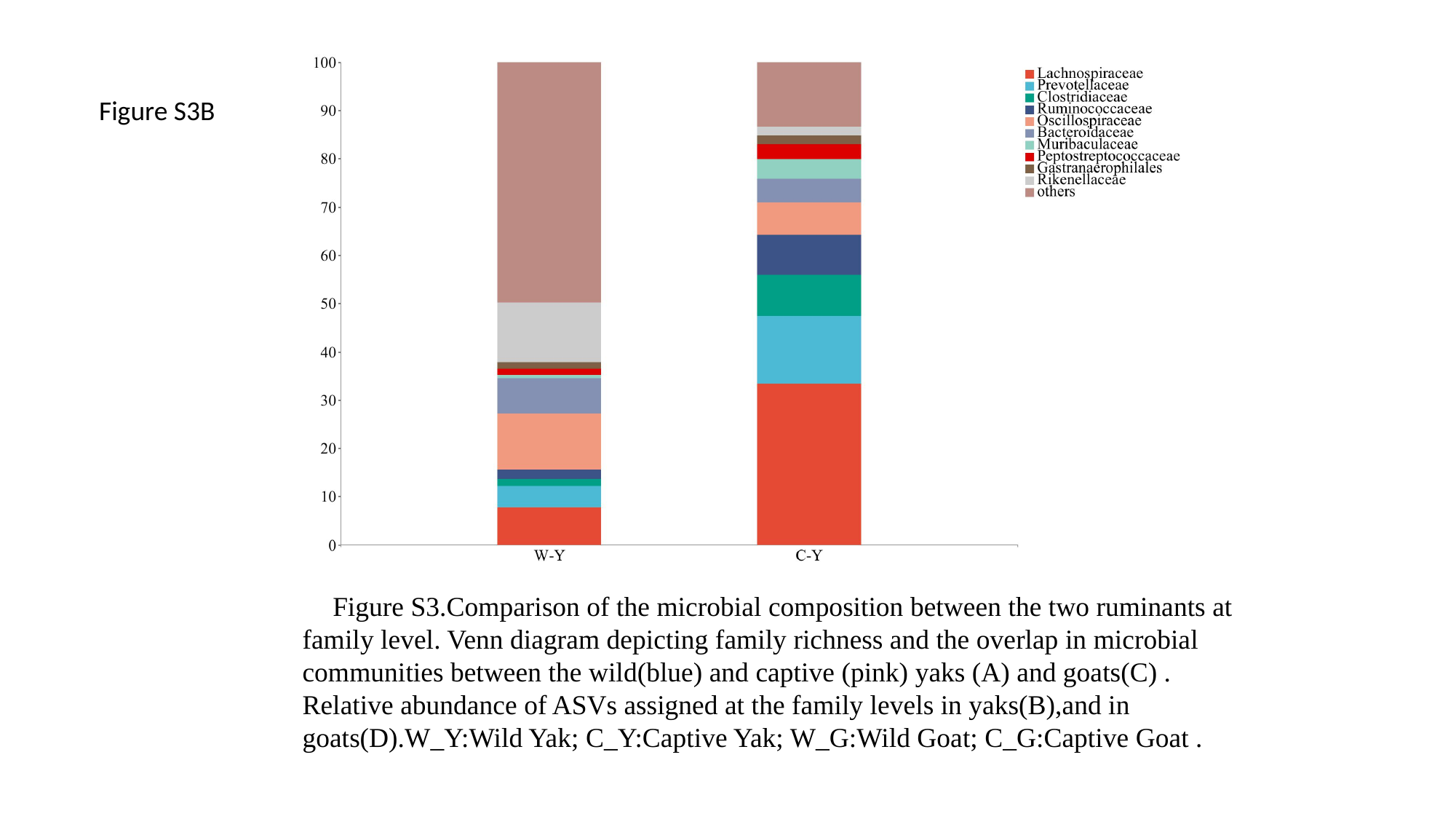

Figure S3B
Figure S3.Comparison of the microbial composition between the two ruminants at family level. Venn diagram depicting family richness and the overlap in microbial communities between the wild(blue) and captive (pink) yaks (A) and goats(C) . Relative abundance of ASVs assigned at the family levels in yaks(B),and in goats(D).W_Y:Wild Yak; C_Y:Captive Yak; W_G:Wild Goat; C_G:Captive Goat .

## Slide 11
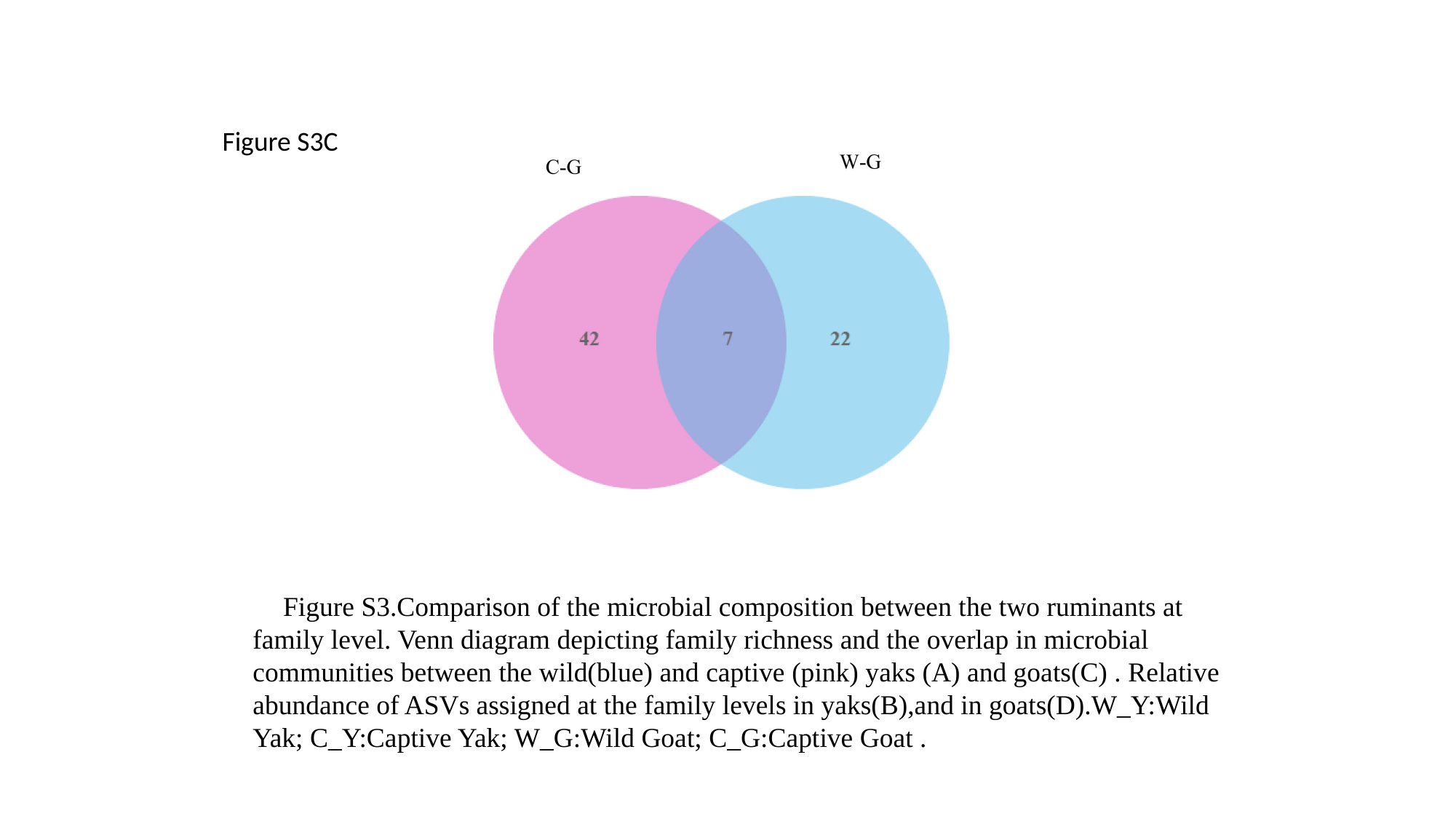

Figure S3C
Figure S3.Comparison of the microbial composition between the two ruminants at family level. Venn diagram depicting family richness and the overlap in microbial communities between the wild(blue) and captive (pink) yaks (A) and goats(C) . Relative abundance of ASVs assigned at the family levels in yaks(B),and in goats(D).W_Y:Wild Yak; C_Y:Captive Yak; W_G:Wild Goat; C_G:Captive Goat .

## Slide 12
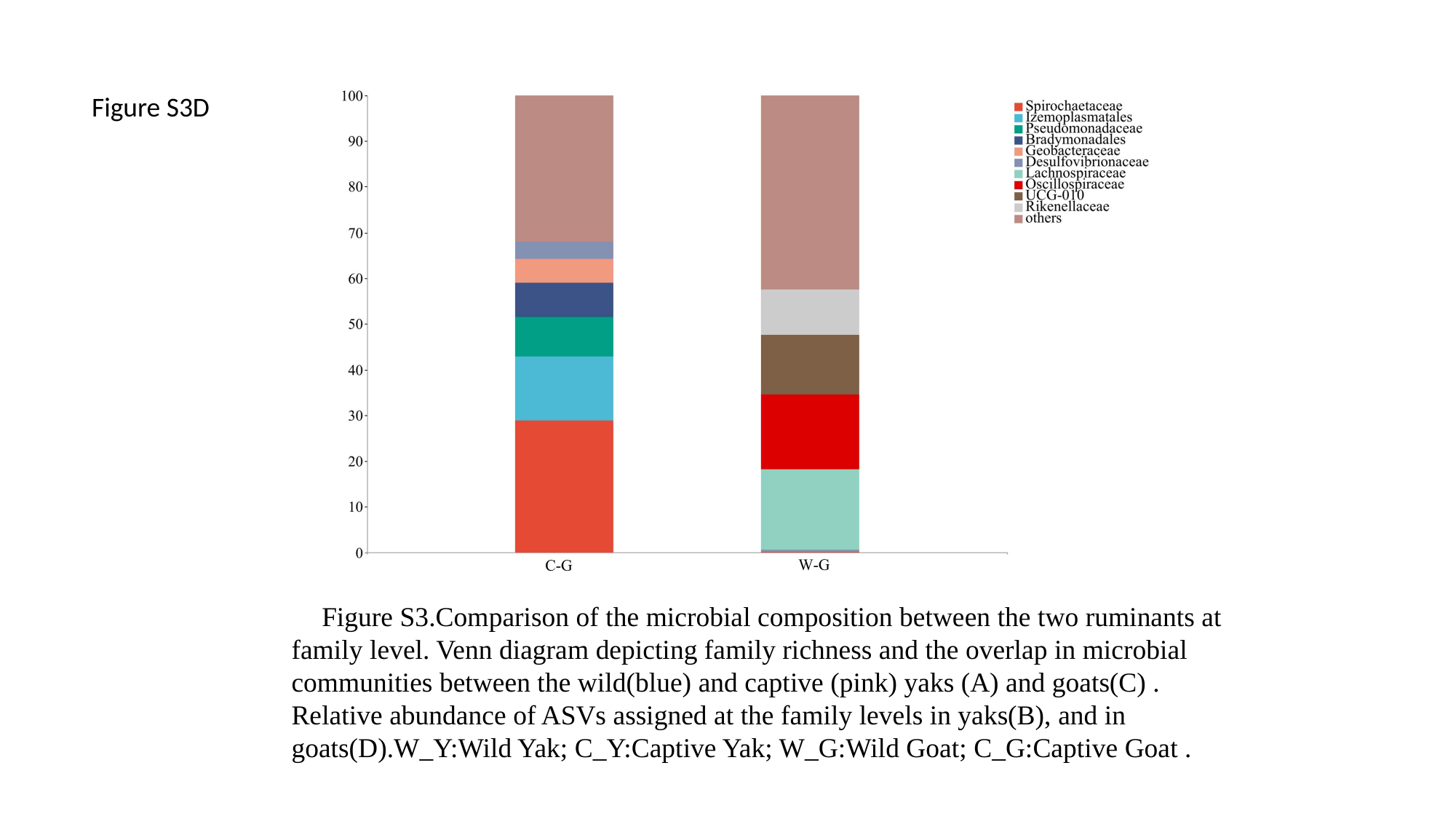

Figure S3D
Figure S3.Comparison of the microbial composition between the two ruminants at family level. Venn diagram depicting family richness and the overlap in microbial communities between the wild(blue) and captive (pink) yaks (A) and goats(C) . Relative abundance of ASVs assigned at the family levels in yaks(B), and in goats(D).W_Y:Wild Yak; C_Y:Captive Yak; W_G:Wild Goat; C_G:Captive Goat .
